# A Special Cranial Nucleus (CSF-Contacting Nucleus) in Primates

**DOI:** 10.3389/fnana.2020.00053

**Published:** 2020-08-12

**Authors:** Si-Yuan Song, Xiao-Meng Zhai, Cheng-Jing Shan, Lei-Lei Lu, Jia Hong, Jun-Li Cao, Li-Cai Zhang

**Affiliations:** Jiangsu Province Key Laboratory of Anesthesiology, Xuzhou Medical University, Xuzhou, China

**Keywords:** CSF-contacting nucleus, primate, rhesus monkey, cerebrospinal fluid, innervation

## Abstract

**Background:**

There is a unique nucleus (CSF-contacting nucleus) in the brain of rat. It has been demonstrated in our previous research. The extraordinary feature of this nucleus is that it is not connected to any parenchymal organ but to the CSF. In primates, however, the presence or absence of this nucleus has not been proven. Confirmation of the presence of this nucleus in primates will provide the structural basis for brain-CSF communication and help to understand the neurohumoral regulatory mechanisms in humans.

**Methods:**

The tracer cholera toxin B subunit conjugated to horseradish peroxidase (CB-HRP) was injected into the CSF in the lateral ventricle (LV) of primate rhesus monkeys. After 48 h, the monkeys were perfused and the brain was dissected out, and sectioned for CB-HRP staining. The CB-HRP positive structures were observed under confocal and electron microscopy. The three-dimensional (3D) structure of the CB-HRP positive neurons cluster was reconstructed by computer software.

**Results:**

(1) CB-HRP labeling is confined within the ventricle, but not leakage into the brain parenchyma. (2) From the midbrain inferior colliculus superior border plane ventral to the aqueduct to the upper part of the fourth ventricle (4V) floor, a large number of CB-HRP positive neurons are consistently located, form a cluster, and are symmetrically located on both sides of the midline. (3) 3D reconstruction shows that the CB-HRP positive neurons cluster in the monkey brain occupies certain space. The rostral part is large and caudal part is thin appearing a “rivet”-like shape. (4) Under electron microscopy, the CB-HRP positive neurons show different types of synaptic connections with the non-CSF-contacting structures in the brain. Some of the processes stretch directly into the ventricle cavity.

**Conclusion:**

Same as we did in rats, the CSF-contacting nucleus is also existed in the primate brain parenchyma. We also recommend listing it as the XIII pair of cranial nucleus, which is specialized in the communications between the brain and the CSF. It is significant to the completing of innervation in the organism.

## Introduction

The animal body consists not only of parenchymal organs, but also of fluids (such as brain interstitial fluid, plasma, and CSF). Physiology studies suggest that the levels and composition of the body fluids are also coordinated under the instructions from the brain (Agnati et al., 2017). The components of the plasma, CSF, and the brain extracellular fluid are different and these fluids are physically separated from each other (Rambeck et al., 2006). How does the central nervous system relay the information to these fluids and functionally modulate their levels and composition remains to be answered.

In the past 30 years, our studies revealed that after injecting peripheral nerve tracer CB-HRP into the rat ventricular system, the tracer is confined to the ventricular wall because of the existence of the brain barriers. The CB-HRP positive neuron cluster is always present at the ventral gray of the lower part of the aqueduct and the upper part of the 4V floor (Zhang et al., 1994, 2003; Lu et al., 2008; Song et al., 2019). Only the neural processes that stretch into the CSF can be labeled. These neurons have a consistent location, form an independent cluster, occupy a certain space, and have a clear boundary with the nearby structures. The CSF-contacting neurons cluster is in accordance with the definition of the nucleus. Therefore, we name it as the “cerebrospinal fluid-contacting nucleus” or “CSF-contacting nucleus” (Song and Zhang, 2018; Song et al., 2019). Studies have confirmed that the CSF-contacting nucleus has broad synaptic and non-synaptic connections with the other neurons and the blood vessels, and has the morphology for sensing and modulating the body fluids (Zhang et al., 2003; Liang et al., 2007; Song et al., 2020a,b).

The discovery of this special structure not only brings new insights to the knowledge of the brain but also describes the “source” regulating the physicochemical property and biological composition of the CSF under different physiological or pathological conditions (Song et al., 2019). Moreover, it provides the morphological evidence of systemic effects after interventions via body fluids (Song et al., 2019). However, these studies were only in rats, the presence or absence of this nucleus has not been proven in primates. The presence of the CSF-contacting nucleus in primates has implications to provide the structural basis for brain-CSF communication and help to understand the neurohumoral regulatory mechanisms in humans.

The present study is the first to describe the CSF-contacting neurons distribution, their nearby structures, 3D spatial morphology, and the synaptic and non-synaptic transmissions between the CSF and the brain parenchyma in primates. This study will provide scientific evidence and theoretical basis of the CSF-contacting nucleus in the primate’s brain.

## Materials and Methods

### Experimental Animals

Adult male rhesus monkeys (average weight of 5.64 ± 2.10 kg) were acquired from the Kunming Primate Research Center of the Chinese Academy of Sciences, license number SCXK (Dian) K2017-0003. All the animal procedures were performed in accordance with the *Guide for Care and Use of Laboratory Animals* and were approved by the Institutional Animal Care and Use Committee (IACUC) of the Kunming Institute of Zoology, Chinese Academy of Sciences. The animals were singly housed (0.80 × 0.80 × 0.80 m) in a controlled environment (temperature: 18–26°C; humidity: 40–70%), with 12 h light/12 h dark cycle.

### Tracer Administration

Monkeys were anesthetized with ketamine (10 mg/kg, i.m.), and the head was fixed on a stereotaxic instrument. 50 μl 30% CB-HRP (Sigma, United States), a specific tracer of the CSF-contacting nucleus through the ventricular system, was injected into the LV according to the stereotaxic coordinates provided by Paxinos et al. (2000). The successful injection was confirmed by extracting the CSF using a microsyringe. After 48 h, the monkeys were perfused.

### Perfusion and Fixation

The animals were anesthetized with ketamine (10 mg/kg, i.m.), the thoracic cavity was opened, and the pericardium was cut to expose the heart. A perfusion needle was inserted into the heart left ventricle. The animals were transcardially perfused with 2000 mL of 0.9% saline to wash out the blood. After, 4000 mL of 4% paraformaldehyde solution was used for fixation. After perfusion, the brain and the spinal cord were dissected out and placed in a 4% paraformaldehyde solution for post-fixation for 48 h at 4°C.

### Tissue Sectioning and Immunofluorescence

After fixation, the brain and the spinal cord were immersed in a 30% sucrose solution until it sank to the bottom. Serial sections (50 μm thick) of the brain and the spinal cord were prepared using a freezing microtome (Leica, Germany). The sections were collected in phosphate buffer saline (PBS) for immunofluorescence staining. The sections were incubated with anti-CB primary antibody (1:600 dilution, List Biological Labs) at 4°C for overnight, followed by incubation with donkey anti-goat Alexa Fluor 594 secondary antibody (1:200 dilution, Life Technologies). Some of the sections were retained for CB-HRP/NeuN double immunofluorescence staining. The CB-HRP stained sections were incubated with anti-NeuN primary antibody (1:50 dilution, Cell Signaling Technology) at 4°C overnight, followed by incubation with donkey anti-rabbit Alexa Fluor 488 secondary antibody (1:200 dilution, Life Technologies). The sections were then mounted in sequence on the slides and counterstained with DAPI before covering with coverslips. The sections were observed and images were captured on a confocal microscope (Zeiss, Germany) using uniform parameters. After capturing the images, some sections were reused for Nissl staining to observe the neural morphology and Nissl body distributions.

### CSF-Contacting Nucleus Mapping and 3D Reconstruction

The neurons in the CSF-contacting nucleus were mapped using the Adobe Illustrator software (Adobe, United States). The outline of the brain sections and the landmarks were illustrated and the neurons in the CSF-contacting nucleus were plotted into separate graphical layers. The brain serial sections and the CSF-contacting nucleus were 3D reconstructed using the Imaris software version 8.4.1 (Bitplane, United States). The “surface” module was used for rendering the brain surface and the CSF-contacting nucleus. The brain surface was colored blue while the CSF-contacting nucleus was colored yellow.

### Visualization of the Synaptic and Non-synaptic Structures of the CSF-Contacting Nucleus in the Monkey Brain

The brain segment of the CSF-contacting nucleus was sliced using a vibratome (Leica, Germany) at 300 μm. CB-HRP staining was performed using the tetramethylbenzidine-sodium tungstate (TMB-ST) method (Gu et al., 1992; Lu et al., 2008). The CSF-contacting nucleus and the ventricle wall were isolated. Transmission and scanning electron microscopy were used to observe the synaptic and non-synaptic connections. (1) For transmission electron microscopy, the sections were immersed in a 2.5% glutaraldehyde solution overnight at 4°C and then postfixed in a 1% OsO_4_ solution for 2 h at 4°C. After, the sections were dehydrated using serial ethanol and embedded in Epon 812 resin. Ultra-thin sections (60 nm thick) were cut using an ultramicrotome (Leica EM UC7), stained with 2% uranyl acetate and lead citrate, and observed under an electron microscope (JEM 1400 Plus, Japan). (2) For scanning electron microscopy, the sections were postfixed in 1% osmic acid for 2 h. After, the sections were washed with distilled water three times (2 min each) and dehydrated using serial ethanol. The sections were then placed in isoamyl acetate and CO_2_ for critical point drying. The sections were placed on the stage using the conducting resin for spraying. The ultrastructure of the ventricle wall was observed using a scanning electron microscope (Teneo VS, United States).

## Results

### CB-HRP Flow Within the Ventricular System and Brain Parenchyma Labeling

**FIGURE 1:**
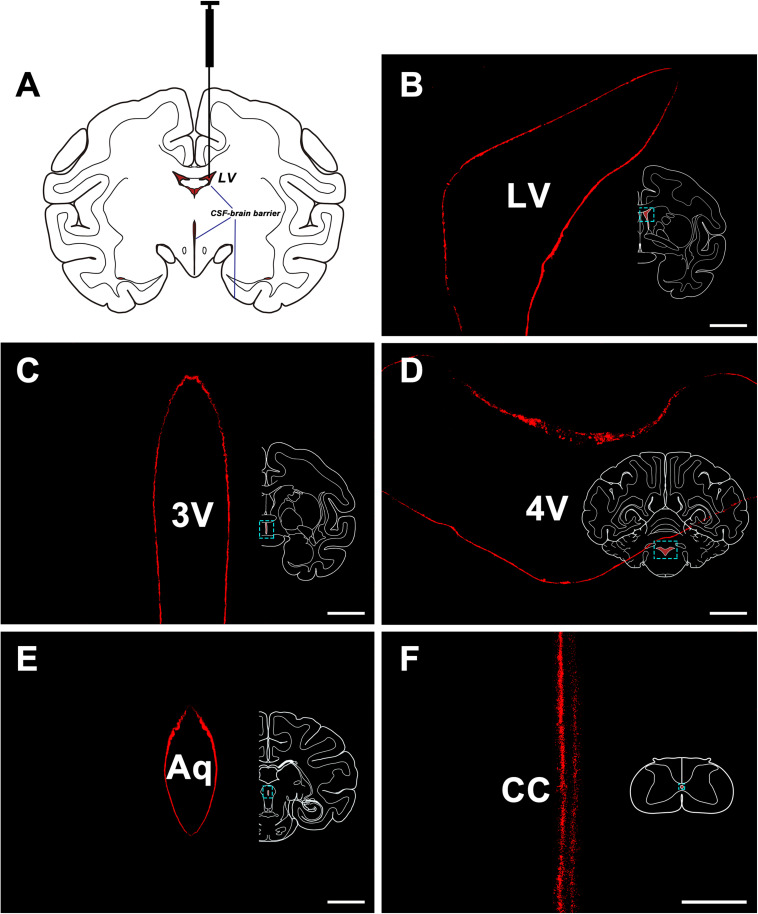
CB-HRP flow in the ventricular system. **(A)** Schematic of the lateral ventricle injection. **(B–F)** CB-HRP immunofluorescence (red) on the ventricular wall. Bar = 0.5 mm **(B–E)** and 0.1 mm **(F)**. LV, lateral ventricle; 3V, 3rd ventricle; Aq, aqueduct; 4V, 4th ventricle; CC, central canal.

The CB-HRP (red-fluorescence) was confined to the ventricular system and formed a clear outline of the lateral ventricle (LV), the third ventricle (3V), the aqueduct (Aq), the fourth ventricle (4V) of the brain, and the central canal (CC) of the spinal cord. The nearby structures are not labeled suggesting that there is no leakage of the tracer (Figure 1).

**FIGURE 2:**
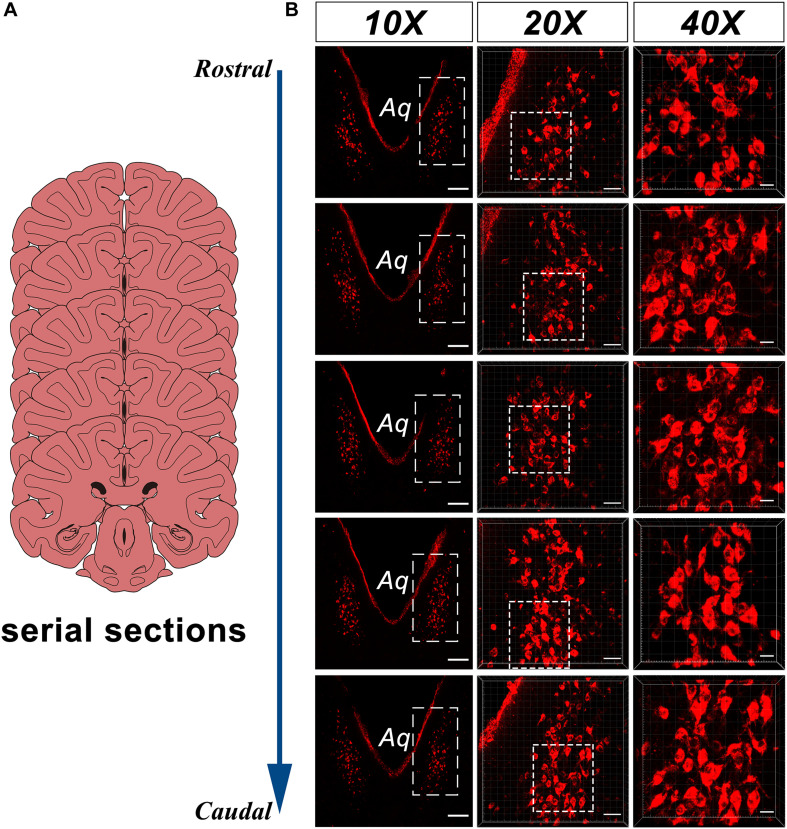
CB-HRP positive neurons of the CSF-contacting nucleus in the parenchyma of the rhesus monkey brain. **(A)** Schematics of the serial sections, **(B)** CSF-contacting nucleus in the serial sections. 10× is the lower magnification. 20× and 40× are the higher magnification of the boxed regions. Aq, aqueduct. Bar = 200 μm in 10×, Bar = 50 μm in 20×, Bar = 20 μm in 40×.

In the caudal part of the ventral gray of the aqueduct and the rostral part of the 4V floor of the monkey brain, a large number of CB-HRP positive neurons is located at consistent regions in the serial sections and form an independent cluster. The somata of these neurons appear to be fusiform or polygonal in shape and are easily distinguished from the nearby, unlabeled structures (Figure 2). These CB-HRP positive neurons are cerebrospinal fluid-contacting neurons.

**FIGURE 3:**
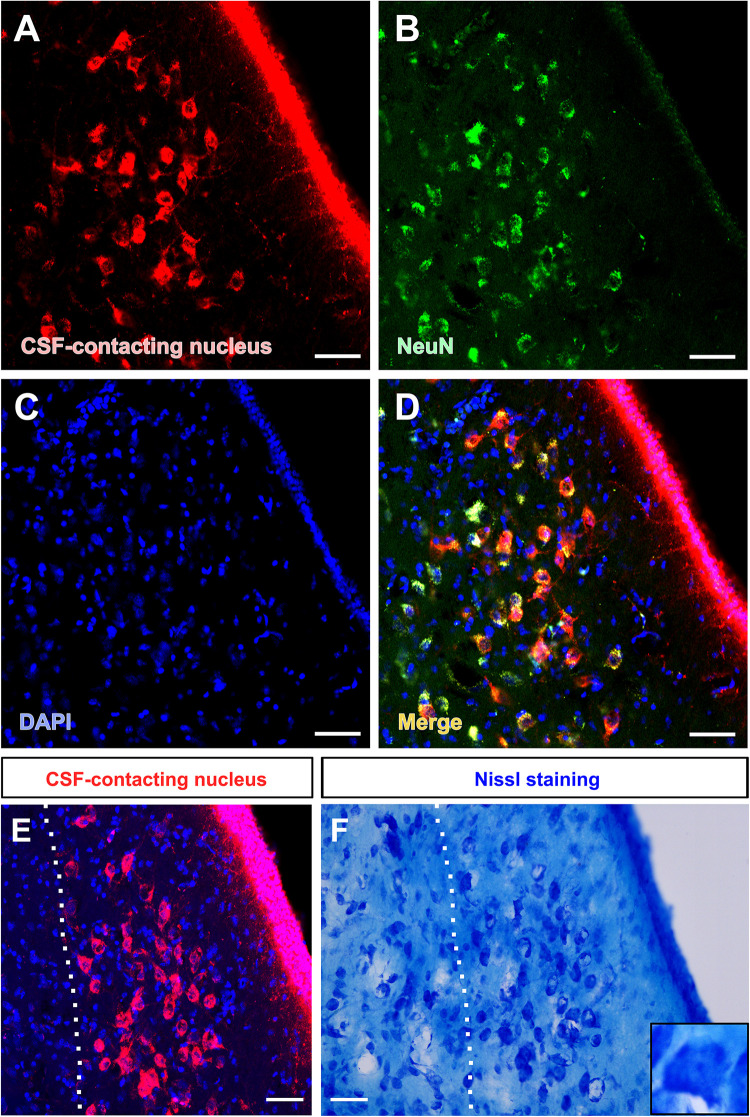
Morphological features of the CSF-contacting nucleus in the rhesus monkey brain under light microscopy. **(A)** Neurons in the CSF-contacting nucleus (red), **(B)** Neuronal marker-NeuN (green), **(C)** DAPI (blue), **(D)** Co-labeling of the CSF-contacting neurons and NeuN (yellow). **(E,F)** The CSF-contacting nucleus **(E)** and its corresponding Nissl staining **(F)**. Bar = 50 μm.

The somata of the CSF-contacting neurons are co-labeled with NeuN, a neuron marker. Further, Nissl staining reveals that the neurons in the nucleus have abundant Nissl bodies. The staining is clear and the CSF-contacting neurons are brightly stained. The somata are slightly larger than the nearby neurons and the nucleolus is clear and evident (Figure 3).

### Position and Adjacency of the CSF-Contacting Nucleus in the Rhesus Monkey Brain

**FIGURE 4:**
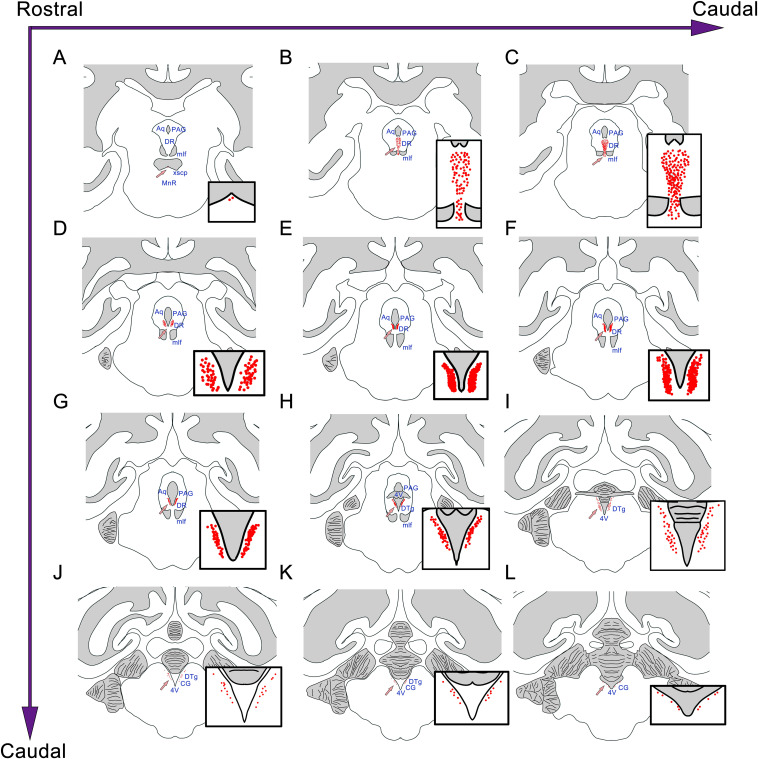
The position and adjacency of the CSF-contacting nucleus in the rhesus monkey brain. **(A–L)** The neurons in the CSF-contacting nucleus are presented as red dots (arrow). The CSF-contacting nucleus position and adjacent structures are shown. Aq, aqueduct; PAG, periaqueductal gray; DR, dorsal raphe nucleus; mlf, medial longitudinal fasciculus; MnR, median raphe nucleus; 4V, the 4th ventricle; DTg, dorsal tegmental nucleus; CG, central gray.

Similar to the distribution of the CSF-contacting nucleus in rats, the nucleus in the monkey brain is located at the ventral gray of the lower part of the aqueduct and the upper part of the 4V floor. The rostral part of the CSF-contacting nucleus is deep in the brain parenchyma and begins ventrally to the decussation of the superior cerebellar peduncle (xscp). The core of the CSF-contacting nucleus is symmetrical and ventral to the aqueduct. In this part, the nucleus is near to mlf, DR, and PAG. The caudal part of the nucleus is located in the ventral gray of the 4V floor, near to the dorsal tegmental nucleus (DTg) and central gray (CG) (Figure 4).

### Three-Dimensional Reconstruction of the CSF-Contacting Nucleus

**FIGURE 5:**
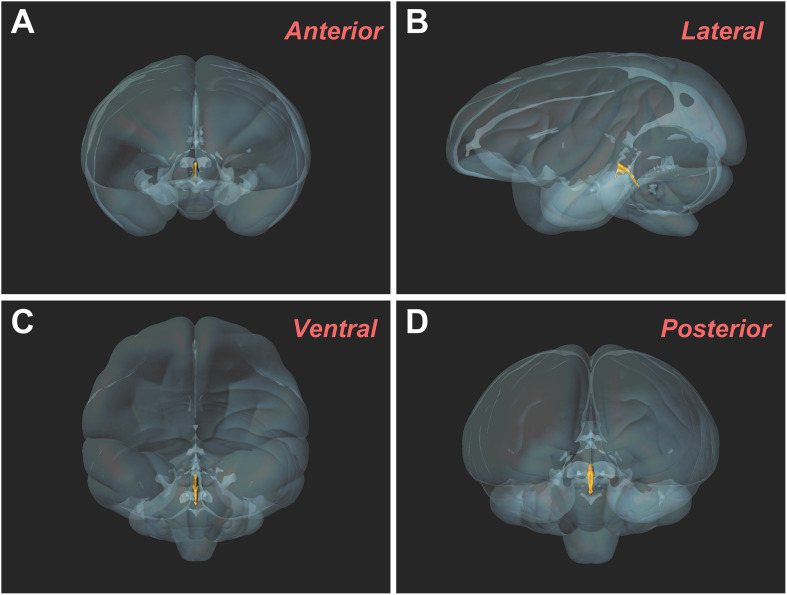
Three-dimensional morphology of the CSF-contacting nucleus in the rhesus monkey brain from different perspectives. **(A)** Anterior view; **(B)** Lateral view; **(C)** Ventral view; **(D)** Posterior view.

The three-dimensional morphology of the CSF-contacting nucleus in the monkey brain was reconstructed using Imaris software. It shows a clear spatial morphology within the brain with definite spatial boundaries and specific location. The nucleus exists independently and consistently in the pons, including the isthmic region. The spatial morphology appears as a rivet-like shape (Figure 5).

### Ultrastructure of the CSF-Contacting Nucleus

**FIGURE 6:**
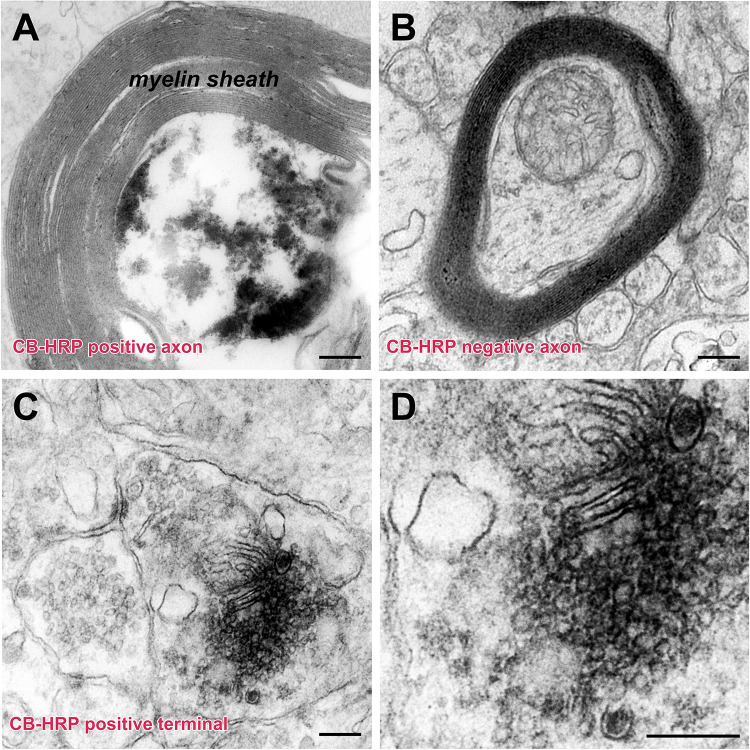
The ultrastructure of the CSF-contacting neuron’s axon and terminal in the rhesus monkey brain. **(A,B)** CB-HRP positive CSF-contacting neuron axon **(A)**, the non-CSF-contacting neuron does not contain positive electron-dense deposits **(B)**. **(C,D)** A CB-HRP positive CSF-contacting neuron terminal containing abundant synaptic vesicles **(C)**; **(D)** is a higher magnification of **(C)**. Bar = 200 nm.

The injected CB-HRP it is transported along the axons of the CSF-contacting neurons. The CB-HRP electron-dense areas can be detected under electron microscopy and the CB-HRP positive axons are covered by a thick myelin sheath. The CB-HRP negative axons show no electron-dense areas. The CSF-contacting neuron terminal contains synaptic vesicles, which are round and flattened. Some of the vesicles have a dense core (Figure 6).

**FIGURE 7:**
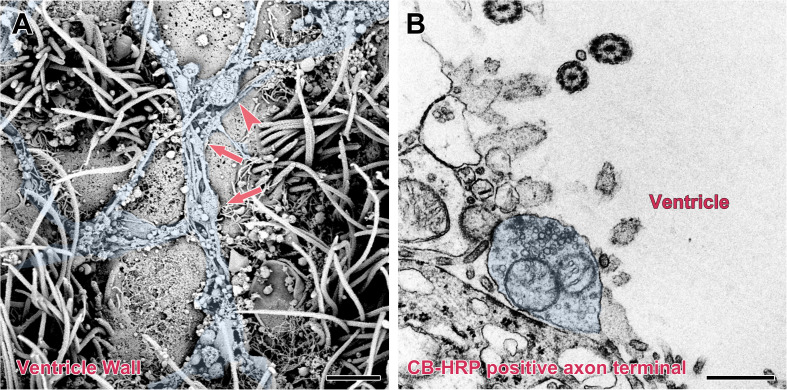
Non-synaptic connections of the CSF-contacting neuron with CSF in the rhesus monkey brain. **(A)** Scanning electron microscopy reveals a large number of neural fibers (blue) with local varicosities (↑) and boutons (▲) located on the surface of the ventricle wall. **(B)** Transmission electron microscopy reveals a CB-HRP positive CSF-contacting neuron terminal (blue) stretching into the ventricle and contacting the CSF. Bar = 200 nm in **(A)** and 500 nm in **(B)**.

The surface of the brain ventricle was observed under the scanning electron microscope. Under the flagellum of the ependymal cells surface, numerous nerve fibers are visible. These nerve fibers have many inflated structures on the segments or terminals. Using transmission electron microscopy, we observed that the nerve fibers are the CB-HRP positive terminals of the CSF-contacting nucleus, which stretch into the ventricular system and contact the CSF forming the non-synaptic connections. The terminals contain many vesicles (Figure 7).

**FIGURE 8:**
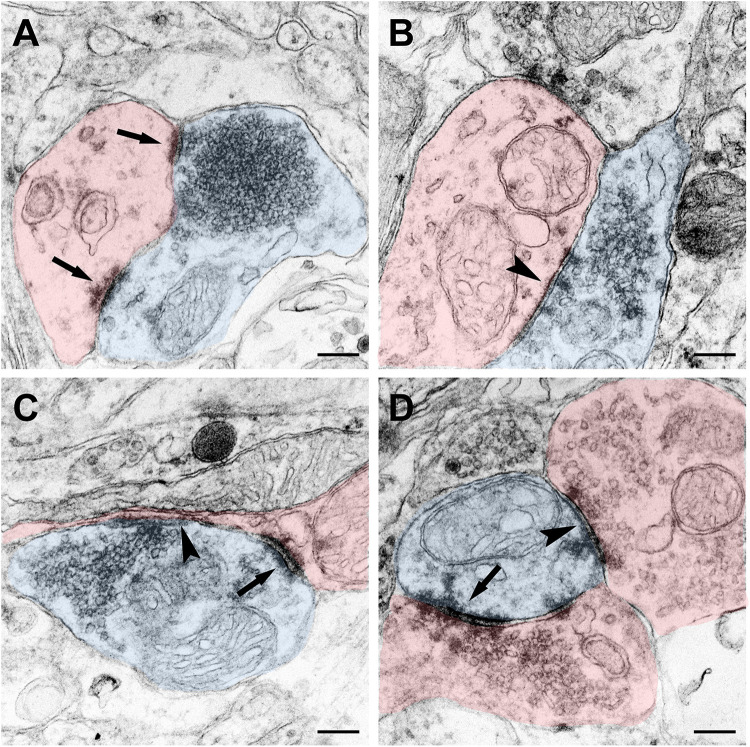
Synaptic connections of the CSF-contacting neuron (blue) with the non-CSF-contacting neuron (red) in the rhesus monkey brain. **(A)** The asymmetrical synapse of the CSF-contacting neuron→non-CSF-contacting neuron (↑); **(B)** Symmetrical synapse of the CSF-contacting neuron→non-CSF-contacting neuron (▲); **(C)** Both asymmetrical (↑) and symmetrical (▲) synapse; **(D)** Asymmetrical (↑) and symmetrical (▲) synapse of the non-CSF-contacting neuron→CSF-contacting neuron. Bar = 200 nm.

The terminal of the CSF-contacting neurons can be a pre-synaptic structure and form Gray I type excitatory (asymmetrical) or Gray II type inhibitory (symmetrical) synapses. Meanwhile, the terminals can also be post-synaptic structures and receive input from other neurons by Gray I or Gray II synaptic forms (Figure 8).

## Discussion

Our previous studies (Zhang et al., 2003; Song et al., 2019) in rodents confirmed that after CB-HRP injection into the CSF of the unilateral ventricular system, it can only flow to the contra-lateral LV, 3V, Aq, 4V, CC of the spinal cord and subarachnoid space. The tracer is localized on the walls of these structures and forms a clear outline. The present study in rhesus monkey confirms that the CB-HRP can only flow within the ventricular system and cannot pass through the CSF-brain barrier and leak into the brain parenchyma. Thus, the neurons whose processes stretch into the CSF can be labeled by CB-HRP. Therefore, the CB-HRP positive neurons observed in our study can only be the CSF-contacting neurons. The reason is that it has been proved repeatedly that there is an objective existence of the CSF-brain barrier.

Naming a nucleus in the brain is a very serious scientific question. It must satisfy strict naming conditions (Moore et al., 2006; Blumenfeld, 2010; Purves, 2012). (1) Similar morphology and functions: In the present study, all of the CB-HRP positive neurons are CSF-contacting neurons whose functions are specified for information connection between the brain and the CSF. (2) Similar location and occupy certain space: The CB-HRP positive neurons are located in the ventral gray of the aqueduct and 4V floor, form an independent cluster and are significantly different from the nearby structures. The three-dimensional reconstruction results show that the entire morphology of the nucleus presents “rivet-like” shape and occupies a certain spatial volume. (3) The distribution characteristics have an evolutionary similarity: In both, rodents as well as in primates, the CSF-contacting nucleus initiates ventral of the xscp and ends at the ventral gray of the 4V floor. The core of the nucleus is located at the ventral gray of the Aq and the nearby structures are DR, PAG, and mlf. The CSF-contacting nucleus in the monkey brain is larger than that in the rats, however, the position and shape are similar. The morphology of the nucleus is highly homologous between the two species. Therefore, we have sufficient evidence suggesting that the CSF-contacting nucleus exists objectively in primates.

The animal body is composed of not only parenchymal organs, but also includes fluids such as brain extracellular fluid, plasma, and CSF. It is believed that the body fluids are modified by the brain for specific functions. For example, neurons release to nearby extracellular fluid through autocrine or paracrine way to complete intercellular communication (Lozic et al., 2018). Hypothalamic pituitary portal system regulates systemic function via releasing neurohormone into blood (Clarke, 2015). However, the cerebrospinal fluid (CSF), an important part of the body, has not been reported to be regulated by a certain nucleus before.

Our study shows that the CSF-contacting nucleus is also existed in primates. Its position is in the pons, including the isthmic region. Although located in the brain parenchyma, it is different from other nuclei in the brain. Its characteristic is that the cell body is in the brain parenchyma, and the processes extend in the CSF, which indicates the characteristics of peripheral nerves similar to 12 pairs of cranial nerves. We recommend listing it as the XIII pair of cranial nucleus, whose basic function is to mediate the information transmission between brain and CSF. This is also very important and necessary in the body, which is of significance to the completing of innervation in the organism. Certainly, deep research is needed to address the further conclusions.

## Data Availability Statement

All datasets presented in this study are included in the article/supplementary material.

## Ethics Statement

The animal study was reviewed and approved by the Institutional Animal Care and Use Committee (IACUC) of the Kunming Institute of Zoology, Chinese Academy of Sciences.

## Author Contributions

S-YS and L-CZ designed the study and prepared the manuscript. S-YS, X-MZ, C-JS, L-LL, JH, and J-LC conducted the studies. All authors read and approved the manuscript.

## Conflict of Interest

The authors declare that the research was conducted in the absence of any commercial or financial relationships that could be construed as a potential conflict of interest.
